# Efficacy and Pharmacologic Data of Second-Generation Tyrosine Kinase Inhibitor Nilotinib in BCR-ABL-Positive Leukemia Patients with Central Nervous System Relapse after Allogeneic Stem Cell Transplantation

**DOI:** 10.1155/2014/637059

**Published:** 2014-06-15

**Authors:** Mark Reinwald, Eberhard Schleyer, Philipp Kiewe, Igor Wolfgang Blau, Thomas Burmeister, Stefan Pursche, Martin Neumann, Michael Notter, Eckhard Thiel, Wolf-Karsten Hofmann, Hans-Jochem Kolb, Stefan Burdach, Hans-Ulrich Bender

**Affiliations:** ^1^Department of Hematology and Oncology, University Hospital Mannheim, Theodor-Kutzer-Ufer 1-3, 68167 Mannheim, Germany; ^2^Department of Hematology and Oncology, Medical Faculty Carl Gustav Carus, Technische Universität Dresden, Fetscherstraße 74, 01307 Dresden, Germany; ^3^Department of Internal Medicine, Klinikum Merseburg, Weiße Mauer 52, 06217 Merseburg, Germany; ^4^Department of Hematology, Oncology and Tumor Immunology, Campus Benjamin Franklin, Charité Berlin, Hindenburgdamm 30, 12203 Berlin, Germany; ^5^Division of Pediatric Hematology/Oncology, Department of Pediatrics, Technische Universität München, Kölner Platz 1, 80804 München, Germany

## Abstract

Central nervous system (CNS) involvement is a severe complication of BCR-ABL-positive leukemia after allogenic stem cell transplantation (alloSCT) associated with fatal outcome. Although second-generation tyrosine-kinase inhibitors (TKI) such as nilotinib have shown activity in systemic BCR-ABL^+^ disease, little data exists on their penetration and efficacy within the CNS. Four patients (3 male, 1 female; age 15–49) with meningeal relapse after alloSCT and subsequent treatment with nilotinib were identified. A total of 17 cerebrospinal fluid (csf) and serum samples were assessed for nilotinib concentration and patient outcome was recorded. Nilotinib concentrations showed a low median csf/plasma ratio of 0.53% (range 0.23–1.5%), yet pronounced clinical efficacy was observed with long-lasting responses (>1 year) in three patients. Comparison with historical data showed a trend towards superior efficacy of nilotinib versus imatinib. Despite poor csf penetration, nilotinib showed significant clinical activity in CNS relapse of BCR-ABL^+^ leukemias. As nilotinib has a high protein-binding affinity, the low-protein concentration in csf could translate into a relatively higher amount of free and therefore active nilotinib in csf as compared to blood, possibly explaining the observed efficacy. Thus, treatment with a 2nd generation TKI warrants further investigation and should be considered in cases of CNS relapse of BCR-ABL-positive leukemia after alloSCT.

## 1. Introduction

In BCR-ABL-positive acute lymphoblastic leukemia (ALL) and advanced stages of chronic myeloid leukemia (CML; accelerated phase, blast crisis (BC)) central nervous system (CNS) involvement is a lethal complication, typically occurring late in the course of the disease, particularly after allogeneic stem cell transplantation (alloSCT). Imatinib was the first specific BCR-ABL tyrosine kinase inhibitor (TKI) to be approved for the treatment of BCR-ABL^+^ ALL and CML and has led to a major breakthrough in the treatment of these malignancies. Nonetheless, 15–20% of patients (pts) with BCR-ABL^+^ ALL or CML-BC develop CNS relapse during ongoing imatinib therapy [1]. This may possibly be attributed to poor CNS penetration and increased cellular resistance mechanisms against the drug such as p-glycoprotein mediated efflux. Standard treatment of CNS relapse is based on intrathecal (ith) and systemic application of cytostatic agents, and/or craniospinal irradiation [2]. Unfortunately, the majority of patients develop subsequent systemic relapse with a very poor outcome of usually less than three months [3] despite initial successful CNS clearance.

Second-generation TKIs such as dasatinib and nilotinib have been approved for the treatment of CML patients who are refractory or intolerant to imatinib.* In vitro* tests of these new TKIs show considerably higher activity in comparison with imatinib, with a 40-fold increased potency for nilotinib and a 325-fold for dasatinib [3].

Moreover, nilotinib and dasatinib were able to overcome imatinib resistance caused by several BCR-ABL tyrosine kinase domain mutations. The potential of these second-generation TKIs in targeting BCR-ABL^+^ CNS disease however is not clear and warrants further investigation. Efficacy of dasatinib in BCR-ABL^+^ CNS leukemia has been demonstrated in an animal model and was furthermore observed in 11 patients. This study also analysed the csf/plasma ratios of dasatinib in 3 patients, showing low levels of dasatinib in csf. For nilotinib, no data on csf penetration and clinical efficacy against CNS relapse of BCR-ABL^+^ leukemia is available so far. Specifically, in pediatric patients very little data has been published on treatment with second generation TKIs at all.

We therefore investigated the clinical activity and csf penetration of nilotinib in four patients with CNS relapse of BCR-ABL^+^ aggressive leukemia after alloSCT.

## 2. Patients and Methods

### 2.1. Patients

Four patients (3 BCR-ABL^+^ ALL, 1 CML-BC; 3 male, 1 female, age 15–49 years) in two centers (Berlin, Munich) treated with nilotinib for CNS relapse after alloSCT were identified. Previous therapeutic regimens include imatinib and dasatinib TKI-therapy for systemic relapse in patients 1–3 while patient 4 had received dasatinib in combination with intrathecal therapy and radiotherapy for meningeal relapse. Investigation of nilotinib csf concentration was performed within a noninterventional diagnostic study, approved by the local ethics committee of the Charite Berlin, Germany (EA4/023/08). Informed consent was obtained from all patients or their legal representatives according to Good Clinical Practice guidelines and in concordance with the Declaration of Helsinki.

### 2.2. Treatment Schedules

Nilotinib 400 mg bid was administered to patients 1–3 while the pediatric patient 4 received an age-adjusted nilotinib dose of 150 mg bid. All patients received additional concomitant antineoplastic treatment (Table 1). Nilotinib was given until obvious disease progression (in patient 1) or detection of the T315I mutation and progression (in patients 2 and 3). Patient 4 is continuing nilotinib treatment at the time of paper submission.

### 2.3. Evaluation of Efficacy

When CNS relapse was suspected, each patient received CNS diagnostics consisting of a cerebral and a spinal magnetic resonance imaging (MRI) scan, csf analysis by lumbar puncture with analysis consisting of cytology including csf differential cell analysis, csf clinical chemistry (glucose, lactate, albumin, and total protein), csf immunophenotyping, chimerism analysis of the csf, and molecular testing for BCR-ABL using PCR for detection of minimal residual disease (MRD).

Further lumbar puncture for assessment of leukemia and nilotinib concentration in csf was performed as clinically indicated. Blood samples were drawn concurrently to calculate csf/plasma ratios for nilotinib. Response assessment was based on analysis of cell count, cytomorphology, immunophenotyping, BCR-ABL quantitative RT-PCR (qPCR) [4], and chimerism analysis [5] in csf. Nilotinib concentration was measured as described previously [6]. Additional cerebral/spinal imaging was performed as clinically indicated.

A csf complete remission (CR) was defined as negative csf cytology, normal csf cell count, immunophenotyping, and cytogenetic and chimerism analysis; a csf partial remission (PR) was defined as negative cytology and csf cell count, but detection of residual disease by either csf immunophenotyping, cytogenetics, or chimerism analysis. Csf minimal residual disease (MRD) negativity was defined as a CR without detection of BCR-ABL amplicons in csf PCR.

## 3. Results

### 3.1. Clinical Data (Table 1)

All four patients experienced CNS relapse after alloSCT. Clinical signs and symptoms that lead to the suspicion of CNS relapse consisted of headache (patients 1–4), facial palsy (patients 1–3), vomiting (patients 1 and 4), and seizures (patient 4). In addition, MRI scans and csf analysis showed the following results for these patients, confirming the diagnosis.

Initial diagnosis of CNS relapse was established by positive cytology, detection of BCR-ABL transcripts, and decrease in donor chimerism in csf with concurrent radiological findings (meningeal enhancement in MRI) in patient 1. In patient 2, a positive csf cytology and immunophenotyping showing lymphatic blasts, a decrease in donor-chimerism, and positivity for BCR-ABL qPCR in csf could be found serologically while MRI scans showed a slight enhancement and an increase in contrast agent uptake. Patient 3 initially had an unremarkable MRI; however in the csf BCR-ABL transcripts could be repeatedly and unequivocally detected, while csf cytology, cell count, chimerism analysis, and immunophenotyping were negative. Patient 4 showed positive radiomorphological results (meningeal enhancement), leukemic blasts in csf cytology, positive immunophenotyping, and BCR-ABL qPCR in csf.

Median time from alloSCT to first CNS relapse was 54 months (range 17–132 months). Median duration of nilotinib treatment was 541 days (range 99–1786 days). Nilotinib treatment was discontinued in patient 1 on day 431 because of progressive disease (intracerebral chloromas), in patient 2 on day 99 because of detection of T315I mutation and systemic disease progression. Both patients died of progressive leukemia. Patient 3, while on nilotinib monotherapy for 847 days, experienced meningeal progression and periodically received additional intrathecal chemotherapy and rituximab from thereon until systemic progression with detection of T315I and F317L mutation. Subsequently TKI treatment was switched after 1786 days of nilotinib treatment to ponatinib. The patient is alive at the time of the paper writing and continuing ponatinib treatment. Patient 4 is currently in remission and continuing nilotinib treatment.

### 3.2. Assessment of Efficacy

Patient 1 achieved a complete remission (CR) with resolution of neurological symptoms, normalization of cytology/immunophenotyping, and restoration of csf donor chimerism; however CNS minimal residual disease (MRD) defined by csf BCR-ABL qPCR positivity persisted. Patient 2 had stable csf cell count and stable csf BCR-ABL transcript levels with minor clinical improvement. Patient 3 showed considerable improvement in symptoms and achieved csf MRD negativity on days 191 and 224 after start of nilotinib treatment. However, minimal amounts of csf BCR-ABL transcripts intermittently reappeared, while csf cytology and chimerism remained negative. When meningeal progression with positive cytology was detected 847 days after start of nilotinib monotherapy intrathecal chemotherapy was added to nilotinib, resulting in intermittent csf MRD negativity until systemic detection of T315I and F317L mutation on day 1786 of nilotinib treatment. Patient 4 achieved a CR in bone marrow and CSF and MRD negativity after three months of treatment with nilotinib and concomitant intrathecal chemotherapy. This patient is continuing nilotinib treatment in complete remission.

In summary, long-lasting responses of more than one year were observed. Patients 1, 3, and 4 had sustained clinical improvement and csf responses while on nilotinib for 431 days (patient 1), 1786 days (patient 3), and >650 days (patient 4), respectively. Both patient 3 and patient 4 are alive; patient 4 is continuing nilotinib treatment.

### 3.3. Comparison of Nilotinib and Imatinib for Duration of Response

**Figure 1:**
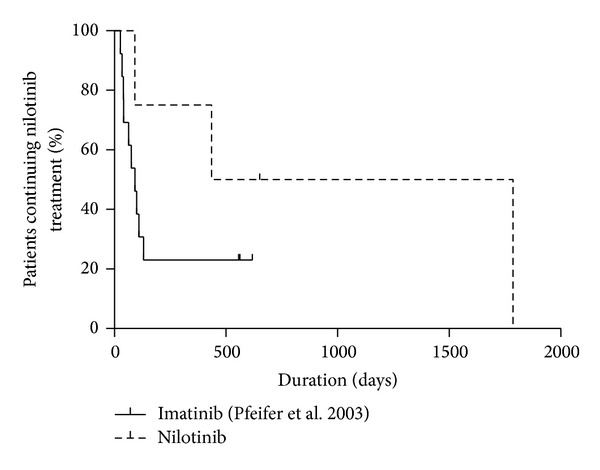
Comparison of nilotinib with imatinib. Kaplan-Meier plot of patients with Ph+ CNS relapse experiencing progressive disease leading to nilotinib discontinuation while on TKI treatment. Data for imatinib was extracted from the historical Pfeifer cohort (Pfeifer et al., [1]) and compared with data from the patients treated with nilotinib in this trial. By trend, patients treated with nilotinib had a longer time to progression, although this was not statistically significant (Chi-square-test 1.9, *P* = 0.17).

Median time to progression (TTP) leading to discontinuation in the nilotinib-treated patients was 541 days with 1 of 4 patients still in remission. Compared with historical data published for imatinib [1] by Pfeifer et al. there was a trend towards an increased TTP for nilotinib treatment (*P* > 0.17, Figure 1).

### 3.4. CSF Pharmacology (Table 2)

In summary 17 csf and corresponding serum samples from 4 patients were assessed for csf penetration of nilotinib. Median csf concentration of nilotinib in patient 1 was 4 ng/mL (*n* = 5, median csf/plasma ratio 0.26%; range 0.23–0.47%), 13 ng/mL in patient 2 (*n* = 3, median csf/plasma ratio 0.83%; range 0.54–1.52%), 7 ng/mL in patient 3 (*n* = 4, median csf/plasma ratio 0.63%; range 0.5–6.5%), and 2.7 ng/mL (*n* = 5, median csf/plasma ratio 0.42%; range 0.26–0.58%) in patient 4. Additionally, a ratio of csf free (unbound) nilotinib and free plasma nilotinib was calculated based on the published nilotinib protein binding of 98% (Table 2).

## 4. Discussion

In CNS relapse of BCR-ABL^+^ leukemia treatment options are limited and survival is poor, especially after alloSCT. The influence of imatinib in treatment and prevention of CNS manifestations seems to be limited, probably due to poor CNS penetration and insufficient antileukemic activity.

To our knowledge, this is the first report on nilotinib csf pharmacology and efficacy in patients with CNS manifestations of BCR-ABL^+^ leukemia after alloSCT. We acknowledge that one limitation of our study is the small number of patients (*n* = 4) with the pediatric patient (patient 4) representing a single case. However, CNS relapse of BCR-ABL^+^ leukemia after allogeneic stem cell transplantation is a rare event with lethal consequences, impeding observation of larger patient cohorts. The number of patients with available pharmacological data (*n* = 4) is similar to that of patients for dasatinib by Porkka et al. (*n* = 3) [7] and the report by Pfeifer et al. for imatinib (*n* = 7) [1].

Csf nilotinib concentrations were within the range of those reported for imatinib [8]. For dasatinib, equally low csf levels were reported by Porrka et al. [7] suggesting that these TKIs have only minimal ability to cross the blood-brain barrier. In dasatinib-treated patients with CNS relapse of BCR-ABL^+^ leukemia the authors found limited csf penetration with low csf/plasma ratios for all 3 patients evaluated. Complete responses were observed in 7/11 patients; the majority received concomitant antileukemic treatment (dexamethasone and intrathecal chemotherapy) but csf MRD analysis for response evaluation was not reported. Additional animal experiments showed superior survival of mice inoculated with CNS leukemia and treated with dasatinib compared to imatinib in the control group. In summary, these combined findings suggest improved efficacy of dasatinib over imatinib in targeting CNS BCR-ABL^+^ leukemia despite poor csf penetration.

Our experience of nilotinib treatment in BCR-ABL^+^-CNS leukemia patients suggests similar clinical activity with sustained responses for more than 1 year (patients 1, 3, and 4) despite low csf drug levels.

In theory, the superior affinity of 2nd generation TKIs to the BCR-ABL-kinase domain could explain these supposedly contradictory findings. The profoundly protein-bound drugs nilotinib and dasatinib have been shown to be active even at subnanomolar concentrations [3];* in vitro* data suggest considerably higher activity compared to imatinib and there might not be a strict dose-response relationship. As csf is a low protein compartment TKI and is more likely to exist as protein-unbound and therefore as active drug. The ratio of free (not-protein-bound) nilotinib in csf relative to free drug in plasma could therefore be increased 5–30 folds compared to the csf/plasma ratio of total nilotinib (Table 2). As meningeal intracellular concentrations could approach systemic intracellular levels, inhibitory concentrations previously published for plasma might thus not reflect those needed for inhibition of bcr-abl in csf. Using patients' csf and plasma protein concentration and an estimation of the amount of free (active) nilotinib the ratio of free to protein-bound nilotinib in the low-protein compartment csf is drastically higher (42–82 times) compared to the high-protein compartment serum. Thus, total nilotinib csf concentrations not reaching the inhibitory concentrations required for systemic activity might nevertheless provide sufficient clinical efficacy in the low-protein compartment csf. This could explain the responses we observed for nilotinib and the results Porrka et al. reported for dasatinib [7]; however as we cannot prove it, further studies should address that issue in more detail.

Recent publications for the TKI erlotinib suggest a clinical benefit in several lung cancer patients with leptomeningeal metastases treated with erlotinib despite poor csf penetration [9], supporting the theory that published inhibitory concentrations for plasma might not reflect those needed in csf.

The long-lasting responses observed in patients 1, 3, and 4 during nilotinib-based therapy clearly suggest an effect of nilotinib and are in stark contrast to the responses published for conventional chemotherapy in relapsed CNS BCR-ABL^+^ leukemia with a poor median overall survival of 2.9 months [10]. Although the addition of 1st generation TKI imatinib does seem to have some effect [11], a direct comparison should be interpreted with care. In our study all patients had developed CNS relapse during imatinib or even dasatinib treatment, and still the majority of patients responded to nilotinib for more than one year. Moreover, the median time to progression in the Pfeifer study was shorter than that observed for nilotinib in our cohort.

While responses of BCR-ABL^+^ ALL to nilotinib monotherapy are rare [12], an early systemic relapse detected only at the molecular level can be successfully managed with TKI-therapy alone [13, 14]. This might explain the observed efficacy of nilotinib as a single agent for more than 2 years even without concomitant antineoplastic treatment in patient 3, whose initial CNS relapse diagnosis was exclusively based on molecular detection of BCR-ABL in csf suggesting a rather small CNS leukemia burden. Furthermore, even open relapse of BCR-ABL^+^ ALL after alloSCT was successfully treated with addition of nilotinib and donor lymphocyte infusions underlining the efficacy of second-generation TKIs in addition to conventional therapy in this clinical scenario [15].

Evaluating the exact influence of TKI-therapy in patients with BCR-ABL^+^ CNS leukemia is difficult as these patients receive a combined treatment strategy consisting of TKI plus other therapeutic measures in routine clinical practice. Moreover, response to TKI-based therapy is additionally influenced by existing or evolving BCR-ABL mutations. Patient 2, who initially responded to a combination of nilotinib and intrathecal chemotherapy, was later found to harbor the T315I mutation. Therefore the observed response was probably exclusively related to concomitant non-TKI treatment as this mutation confers resistance to nilotinib. Compared to the other three patients, however, treatment response in this patient was short lived (99 days versus 431+ days) underlining the necessity of effective BCR-ABL suppression. The pronounced influence of mutation status is also highlighted by the clinical course of the other patients. Patient 1 experienced meningeal progression during dasatinib and INNO406 treatment, both drugs supposedly penetrating into the CNS [7, 11]. Despite progression under these treatment regimens, an excellent response to nilotinib was observed. In fact, the V299L mutation was later detected in this patient and might explain this clinical course, as this mutation confers a poorer efficacy for dasatinib [16]. The outcome of patient 3, who developed a systemic relapse with T315I and F317L mutations also demonstrates the possibility of evolving resistant mutations under constant selective pressure of long-lasting TKI treatment. Based on our data, switching therapy to a different 2nd generation TKI in BCR-ABL^+^ CNS disease should be strongly considered if the patient fails to respond, a strategy that has been proven effective in systemic treatment [17].

## 5. Conclusions

In summary, our findings suggest relevant activity of nilotinib in BCR-ABL^+^ CNS relapse after alloSCT despite limited csf concentrations which may be attributed to the low-protein environment of csf. As treatment options are limited for these heavily pretreated patients with poor prognosis, clinical trials should further elucidate the role of different TKIs in this clinical setting. In light of our data, the addition of a 2nd generation TKI should be considered in BCR-ABL^+^ meningeal relapse after allogeneic stem cell transplantation.

## Figures and Tables

**Table 1:** Patient characteristics and clinical data.

Patient	Age [year]	Underlying disease	Therapy prior to CNS relapse	Time of CNS relapse [months after diagnosis]	Symptoms at CNS relapse	Diagnosis of CNS relapse	Concomitant anti leukemic treatment for CNS relapse [d]^¥^	BCR-ABL mutation detected	Duration of nilotinib monotherapy [d]	Best response in CSF	Duration of nilotinib administration (mono + concomitant therapy)	Type of relapse/progression
1	38	CML-BC	AlloSCT, DLIs, imatinib, and dasatinib	132	Headache, vertigo, and facial palsy	Clinical signs and symptoms, csf cytology, csf immunophenotyping, csf BCR-ABL qPCR, and decrease in csf donor chimerism	Intrathecal triple therapy∗[d−100, d−98, d−95, d−91], INNO406 360 mg tid, [d−66 to d−53], Donor lymphocytes [d−28, d−23, d−14, d−7, d0, d+7, d+14, d+25], 2000 mg/m^2^ Cytarabin i.v. [d−35 to d−34]	V299L	406	CR with MRD positivity	431	Systemic (intracerebral chloromas)

2	19	c-ALL	GMALL-protocol, alloSCT, and dasatinib	17	Headache and anisocoria	Clinical signs and symptoms, csf cytology, csf BCR-ABL qPCR, and decrease in csf donor chimerism	Intrathecal triple therapy∗ [d+10, d+29, d+52, d+78], 375 mg/m^2^ rituximab i.v. [d+30, d+38, d+53] 2 mg vincristine i.v. [d+29, d+56, d+78] 4 mg dexamethasone bid. [d+52 to d+83]	T315I	NA^*£*^	SD	99	Systemic (bone marrow)

3	49	c-ALL	GMALL-protocol, alloSCT, and dasatinib	28	Facial palsy headache	Clinical signs and symptoms, csf BCR-ABL qPCR, and positive csf cytology on 2nd relapse	Intrathecal triple therapy∗ [d+848, d+851, d+855, d+858, d+864, d+871, d+878, d+892, d+1310, d+1421, d+1531, d+1544, d+1547, d+1551] Rituximab 20 mg i.th [d+1320, d+1334, d+1341, d+1356, d+1369, d+1382, d+1554, d+1560, d+1567, d+1618, d+1671, d+1740]	T315I, F317L	847	MRD negativity	1786	Systemic (peritoneum)

4	15	c-ALL	ALL-BFM2000, alloSCT. DLI, Ifn, GM-CSF, imatinib, and ALLRez2002	80	Headache, vomiting, and seizures	Clinical signs and symptoms, csf BCR-ABL, and BM BCR-ABL	Dasatinib [d−60–182] Radiotherapy [d−32–45] Intrathecal triple therapy^€^ [d−60]	None	NA^*£*^	MRD negativity	650+	In remission

PR = partial response, SD = stable disease, CR = complete remission, NA = Not applicable, c-ALL = common ALL, DLI = donor lymphocyte infusion, Ifn = Interferon; GMALL-Protocol: Polychemotherapy induction, consolidation and postremision protocol of the german ALL study group for adults; BFM2000: Polychemotherapy induction, consolidation and postremision protocol of the german ALL study group for pediatric patients; i.v.: intravenous; i.th = intrathecal,

^¥^relative to start of nilotinib treatment; ∗consisting of 15 mg MTX, 40 mg Cytarabin and 4 mg dexamethasone; ^€^consisting of 12 mg MTX, 30 mg Cytarabin and 10 mg prednisone at 4-week intervals for one year and 6-week intervals after one year; ^*£*^nilotinib applied with periodic concomitant anti-leukemic treatment.

**Table 2:** Pharmacokinetic data.

Patient	Nilotinib csf concentration [ng/mL], (range)	Nilotinib plasma concentration [ng/mL], (range)	Nilotinib csf/plasma ratio [%], (range)	Csf protein concentration [g/L]	Plasma protein concentration [g/L]	*Free* nilotinib in plasma concentration [ng/mL]∗	Nilotinib csf concentration/*free* nilotinib plasma concentration [%,]∗
1	4 (3.6–15)	1640 (1544–1736)	0.26 (0.23–0.84)	0.49 (0.31–0.75)	76 (69–77)	32.8	12
2	13 (4–18)	955 (734–1176)	0.83 (0.54–1.52)	0.40 (0.34–0.89)	65 (56–69)	19.1	68
3	7 (6–9)	1159 (1082–1237)	0.63 (0.5–0.79)	0.50 (0.28–0.67)	59 (56–67)	23.2	30
4	2.7 (1.5–4.8)	682 (430–1034)	0.42 (0.26–0.58)	0.23 (0.19–0.31)	71 (62–78)	13.6	20

All values are given as median.

∗Unbound (=free) nilotinib concentration calculated by using published nilotinib protein binding of 98%.
